# A systematic review and meta-analysis of patient education in preventing and reducing the incidence or recurrence of adult diabetes foot ulcers (DFU)

**DOI:** 10.1016/j.heliyon.2018.e00614

**Published:** 2018-05-02

**Authors:** P. Adiewere, R.B. Gillis, S. Imran Jiwani, A. Meal, I. Shaw, G.G. Adams

**Affiliations:** a The University of Nottingham, School of Health Sciences, B Floor, South Block Link, Queen's Medical Centre, Nottingham, NG7 2HA, UK; b The University of Nottingham, Faculty of Social Sciences, University Park, Nottingham, NG7 2RD, UK

**Keywords:** Public health, Health sciences

## Abstract

**Background:**

The World Health Organization (WHO) states that diabetic foot ulcers (DFU) are associated with disability, death among patients with diabetes and substantial costs, if not prevented or managed effectively. The aim here is to examine the effectiveness of patient education in preventing and reducing the incidence or recurrence of adult DFU and amputation.

**Methods:**

A systematic review and meta-analysis of randomised clinical trials (RCTs) in adults aged 18+ who have diabetes mellitus (type 1 or type 2) or DFU. CINAHL, EMBASE, MEDLINE, PSYCINFO, Cochrane Library and Evidence-Based Nursing, National Library for Health, Medica and Google Scholar were searched. Only English language studies were considered. Databases were searched from their inception to September 2017.

**Findings:**

Six RCTs met the inclusion criteria. Only five RCTs reported on the incidence of DFU whilst only two reported on amputation rates. There was no advantage of combining different educational approaches in preventing/reducing DFU, relative risk (RR) of 0.50 (95%CI 0.21, 1.17) (P = 0.11). Two RCTs based on foot care education alone were compared with usual care; the result showed a non-significant effect (P = 0.57) with high heterogeneity of 77%.

Analysis based on intensive versus brief educational approach showed a statistically reduced risk of incidence of DFU in the intervention group when compared to the control group; (RR, 0.37, 95%CI 0.14, 1.01) (P = 0.05) with high heterogeneity of 91%.

**Interpretation:**

The intensive educational intervention was associated with reduced incidence of DFU.

## 1 Introduction

With 422 million people diagnosed with diabetes mellitus (DM) in 2015, the number is projected to rise to 642 million by 2040 (International Diabetes Federation (IDF), 2017). This has largely been attributed to unhealthy lifestyles, population growth, ageing, globalisation and urbanisation (World Health Organization (WHO), 2017, World Health Organization(WHO), 2016; Goie and Naido, 2016).

DM affects the nervous system and can lead to peripheral neuropathy and autonomic malfunction (Yazdanpanah et al., 2015). Sensory symptoms are much more prominent than motor in typical diabetic neuropathy (Sung et al., 2017). Sensory neuropathy results in a reduced or loss of sensation leaving the foot vulnerable to trauma contributing to skin breakdown and foot ulcer formation (Boike et al., 2017), while motor neuropathy leads to deformity and structural changes in the foot. These structural deformities continuously increase mechanical stress/pressure on these digital contractures with the cells of the foot reacting to abnormal pressure by increasing cell keratinisation predisposing to DFU (Arosi et al., 2016). Autonomic neuropathy aberrations and damaged nerves compromise the circulation system and the sweat glands (Turns, 2015) resulting in fissures due to decrease in sweat and callus formation as a result of extrinsic stress (Edmonds and Foster, 2014). Simultaneously, poor vascular perfusion and immunity may impede wound healing and increase the risk of infection (Arosi et al., 2016). Neuro-osteoarthropathy and high plantar pressure (van Netten et al., 2016; Yazdanpanah et al., 2015) are also additional concerns with the rate of lower limb amputation in people with DM being 10–30 times higher than people without (Aalaa et al., 2012). According to The International Working Group on the Diabetic Foot (2016), globally, every 20 seconds a leg is amputated due to DM with the lifetime incidence of DFU in patients with diabetes between 15-20% and the possibility of recurrence between 30-40% within the first year (van Netten et al., 2016; Armstrong et al., 2017).

Many patients with DM lack foot care knowledge but patient participation is a key determinant of successful management of disease, especially long-term ill health (Ritsema et al., 2014). Health education (Dorresteijn and Valk, 2012) is a key resource in helping patients understand and engage in the management of their health conditions (Green-Morris, 2014) and a daily foot check the most common preventative measure for DFU (Jeffcoate et al., 2011; Alexiadou and Doupis, 2012). Despite the widely advocated use of educational interventions in DFU prevention, there are few systematic reviews (Dorresteijn et al., 2014; Singh et al., 2005; Majid et al., 2000; Mason et al., 1999) with most concentrating on uncontrolled studies; although Dorresteijn et al. (2014) cited insufficient evidence to demonstrate the effects of education on DFU prevention (Nolan et al., 2011).

Here we examine the effectiveness of patient education in preventing and reducing the incidence or recurrence of foot ulcers in adult with diabetes. In order to achieve this we 1) assessed the incidence of DFU post-educational intervention and 2) amputation rates post-educational intervention.

## 2 Methods

### 2.1 Search strategy and selection criteria

The following electronic health databases were searched: MEDLINE, EMBASE, Cumulative Index to Nursing and Allied Health Literature (CINAHL), PSYCINFO, Cochrane Library and Evidence-Based Nursing. An additional search was performed on subject gateways (Nursing portal, National Library for Health, Medica and Google Scholar); bibliographies of all relevant retrieved studies and text books relevant to the research questions. Searches were carried out by two researchers GGA and PA and conflicts over inclusion resolved through discussion.

## 3 Study inclusion and exclusion criteria

Participants included those aged 18 and above who have DM, type 1 DM (T1DM), type 2 DM or those with DFU. The intervention was focused on patient education and the incidence of DFU post-educational intervention in addition to amputation rates post-educational intervention. Only those studies carried out and published in English were accepted for inclusion.

In terms of exclusion criteria, studies which focused on conditions associated with DFU among children or animals were excluded from the study. Other ulcers were excluded because of different underlying aetiologies.

The methodology adopted for this study includes a review of relevant literature obtained through search strategies based on appropriate inclusion and exclusion criteria using Population, Intervention(s), Comparator(s), Outcome and Studies/Settings (PICOS) or Type of Study (T).

### 3.1 Selection of studies

**Fig. 1:**
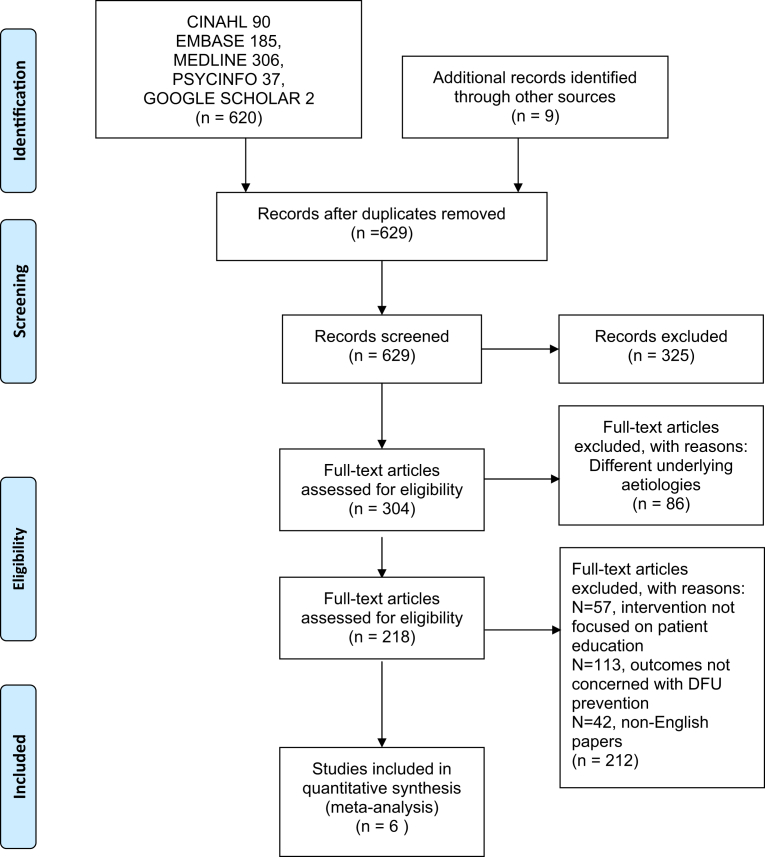
Records identified through database screening.

Results from different databases and sources were combined and titles and abstracts examined to remove irrelevant studies. Studies published in duplicate were included once. Full texts of potentially appropriate studies were examined using the inclusion and exclusion criteria. The search was first conducted on 11th March 2017 and updated on the 5th September 2017. The selected RCTs included totalled of 6 papers from electronic health databases (Fig. 1). This study approach is based on Preferred Reporting Items for Systematic Reviews and Meta-Analyses (PRISMA) guidelines (Moher et al., 2009).

### 3.2 Data extraction

The data extraction was carried out using a pre-piloted data extraction tool formed by Higgins and Deeks (2009).

### 3.3 Appraising the quality of the included studies and risk of bias

The quality of the RCTs was assessed for the risk of bias based on the following six domains (randomisation method, allocation concealment, blinding, incomplete outcome data, selective outcome reporting and other bias).

Meta-analysis was used to analyse DFU and amputation rates. A Critical Appraisal Skills Programme (CASP) tool was used as a means of assessment. Quality assessment was carried out by two researchers GGA and PA.

### 3.4 Measures of treatment (intervention) effect

For dichotomous outcomes (DFU or amputation rates) after educational intervention, the result was presented as a relative ratio (RR) with corresponding 95% confidence intervals (CI).

### 3.5 Data analysis

For each study, the data for the events (DFU and amputation rates after educational intervention), and the number of participants were pooled. In addition, a weighted average of the intervention effects was preserved to ensure larger studies were given more weight in meta-analysis when compared with smaller studies. For Rönnemaa et al. (1997) callosities located in the calcaneal region was used to calculate the incidence of DFU. All analyses were carried out using the Review Manager 5.3 (RevMan).

### 3.6 Assessment of heterogeneity

The Chi-squared (Chi^2^) was used to assess the significance for heterogeneity or as a measure of variability between studies. It also allows for assessment to check whether the observed difference in the treatment effects are due to chance alone with a low P-value indicating presence of heterogeneity.

### 3.7 Presentation and interpretation of results using meta-analysis

The meta-analyses results are presented using a forest plot produced using Review Manager 5.3 (RevMan) (The Cochrane Collaboration, 2011).

### 3.8 Risk of bias of included studies

Studies were assessed using The Cochrane Collaboration tool for assessing risk of bias was used to assess the included studies (Higgins et al., 2011). The risk of bias of most included RCTs was high, except Lincoln et al. (2008).

In addition, Caldwell's critical appraisal tool was used for quantitative studies to assess the quality of the studies. The included studies were numbered 1–6 in accordance of the most recent. The studies were appraised by answering yes or no to the critical appraisal tool questions. The 12th question was divided into two parts so that it could be answered clearly to reflect the population under study. The final question in the critical appraisal tool will be discussed individually, due to the answer being neither a distinctive yes nor no. Studies answering yes to 14 or more of the 16 questions were selected for inclusion in the review.

## 4 Results

### 4.1 Description of included studies

The 6 RCTs were published between 1986 – 2017 (Table 1). The studies were carried out in different countries and conducted in different health settings. All subject participants (both males and females) were aged 18 years and above with DM, T1DM, T2DM or DFU and the median time for follow-up of the studies was within 6 months.

**Table 1:** Characteristics of included studies.

No	Author/Year	Country	Study setting	Study participants/Sample size	Characteristics of education intervention	Duration and follow-up strategies	Outcome/Findings
1	Monami et al. (2015)	Italy	Outpatient clinic	120(intervention 60 versus control 60) participants aged 18 and above with T2DM with at least three of the criteria (neuropathy, previous DFU, foot abnormalities at risk in the opinion of the investigator).	Intervention group: They had 2-hour (90 minutes interactive session on practical exercise on behaviour modification and 30 minutes face to face lessons on risk factors in developing DFU) education program in a group session of 5–7 participants. The intervention provided a physician for 15 minutes and a nurse for 105 minutes. PIN (Patient Interpretation of Neuropathy) questionnaire was administered before and at the end of the educational session Control group: They were given brief leaflet information on foot ulceration as suggested by local protocols.	Planned at 3 and 6-month follow-up visit	**P-**foot ulcer **S**-foot care knowledge. Due to the high difference in outcome between the two groups, the study was prematurely terminated. Foot ulceration. RR 0.08(0.00–1.34) Though participants' knowledge about foot care improved (intervention 20 versus control groups 23, P˂0.001). 6 participants developed ulcers during the 6 months follow-up (10% versus 0%, P = 0.012).
2	Gershater et al. (2011)	Sweden	Multidisciplinary foot clinic	131(intervention 61 versus control 70) participants aged 35–79 years with DM, neuropathy and prior DFU accepted the invitation to participate in the study. 67.1% with T2DM, 32.9% with T1DM. Time of evolution of DM not provided.	Intervention group: They had usual care + 1-hour group sessions on foot ulcer conducted by a nurse. In all 14 group sessions: 10 sessions for men, 4 for women with 2–5 participants in each group. Each subject participated once in the group session. Control group: Footwear with soles. Standard information was based on international consensus on diabetic foot provided by diabetes specialist nurse.	After 6 months of post enrolment the feet of all subjected regardless of intervention were evaluated using the Wagner classification where level 0 indicates feet with no ulcer while level ≥ 1 indicates ulcer.	**P**- Incidence of new ulcers 42% of 98 participants who completed the follow-up developed new foot ulcers (intervention 19 versus 22 in control group). The result showed that the intervention was not effective in reducing the incidence of DFU.
3	Lincoln et al. (2008)	United Kingdom	Secondary outpatient clinic: specialist foot clinic	172 participants with DM and a newly healed foot ulcer were randomised into groups (intervention 87 and control 85). Baseline risk of ulceration include loss of stimulus perception of 10g monofilament were present in the participants (intervention 47% versus control 42%); loss of vibration perception intervention 68% versus control 62% Loss of neuro tip perception: intervention 35% versus control 36%	Intervention group: They had an hour structured foot care education session by a researcher during home visit and reinforced by a single telephone call after 4 weeks of the educational session to keep up with the program content (causes of foot ulcers and evaluation of foot wear). In addition, handouts were given comprising information on the key causes of foot ulcer and how to avoid them. The control group had same handouts.	After 6 and 12 months post enrollment. 168 participants completed follow-up for primary outcomes while 138 participants completed a year follow-up for the subordinate outcomes. Outcomes assessment were done using medical records and supplemented by using questionnaires with multiple choice answers.	**P**-Ulcer incidence (recurrence), amputation rate. **S-** Participants' behaviour assessment score There was significant improvement in foot care behaviour at 12 months in the intervention group when compared with the control (P = 0.03). However, there was no evidence that the target education had clinical benefits for diabetic participants enrolled in an educational program.
4	Rönnemaa et al. (1997)	Finland	Community -based care	530 participants with DM randomised (intervention 267 versus control 263). Information regarding foot ulceration at baseline was not provided.	Intervention group: They had 45 minutes intensive education program comprised of discussions on proper foot wear/hygiene, combined with podiatry care. Control group: They had written information on foot care only	1 and 7 years post enrolment; 459(intervention 233 versus control 226) finalised one year of follow-up. 332(intervention 169 versus 163) completed seven years of follow-up.	**P-** amputation rate, ulcer incidence **S**- Foot care knowledge, callus development, assessment of behaviour scores. The result showed an increase in foot care knowledge in the intervention group when compared with the control group after 12 months of follow-up (P = 0.004). There were no effects on patient education on DFU and amputation rate
5	Malone et al. (1989)	USA	Secondary outpatient care, podiatric or vascular surgery care	227 participants with DM, DFU or prior amputation. 203 were included in the study (intervention 103 versus control 100)	Intervention group: They had an hour group education given by a podiatrist using slides that contain infected DFU and amputated limbs and simple instructions on foot care. In addition, routine patient education (diet teaching on diet, weight, exercise and medication was given). Control group: They had routine patient education.	Intervention median time was 13.2 months v control median time 9.2 months. 182(intervention 90 versus control 92) participants completed follow-up	**P**- Incidence of ulcer infections and amputation rates. **S** –No information provided The outcome of the study was reported per limb (n = 354) instead of per participant. The findings showed a marked reduction of ulcer incidents (intervention 8 versus control 28), amputation rates (intervention 7, control 2) observed in intervention. Relative Risk (RR) for foot ulceration was 0.31 95% CI (0.14–0.66) and RR for amputation was 0.33 95%CI (0.15–0.76).
6	Bloomgarden et al. (1987)	USA	Diabetes Clinic	Originally 749 with DM (unclear on the type) being treated with insulin were recruited for the study. 345 (intervention 165 versus control 180) out of 749 participants consented to participate. Information regarding foot ulceration at baseline was provided: 146 (intervention 83 versus control 63) participants had no foot lesions at initial evaluation 100 participants (intervention 37 versus control 63) had callus, foot nail problems, fungal infection while 20(intervention 7 versus control 13) had an ulcer or amputation on initial evaluation	Intervention group: They had 1: 9 group patient education sessions involves using film, card games and individual instructions by the health professionals (nurse educator and nutritionist). 1 group session of patient education entails education on foot care and skin hygiene while others focus on understanding the basics of diabetes, its complication, insulin administration and balanced nutrition, Educational adherence group session:82(50%) of intervention group completed at least 7 or more sessions Control group: They had usual care and the content of the care was not specified.	Intervention 1.6 ± 0.3 years versus control 1.5 ± 0.3 years; 266 participants completed follow-up: intervention 127 versus control 139	**P-**Ulcer incidence or amputations. **S-** Calluses, foot/nail problems and infection due to fungi and assessment score based on behaviour. There were no significant effects of patient education as observed in the incidence of foot ulceration and amputation and even on callus formation, nail dystrophy and fungal infection.

### 4.2 Addressing the six domains in assessing the quality of the included studies

#### 4.2.1 Randomisation generation

4 studies out of the 6 included studies have clear method of randomisation

• Via computer generated lists held by an independent randomisation centre (Monami et al., 2015; Gershater et al., 2011).
• Randomisation was generated using a computer before commencing the study (Lincoln et al., 2008).
• Participants were randomised into intervention and control groups based on the odd or even last digit of their social security number (SSN) (Malone et al., 1989).

2 studies had unclear randomisation method.

Information was not provided (Rönnemaa et al., 1997; Bloomgarden et al., 1987).

#### 4.2.2 Allocation concealment

4 studies have a clear method of allocation concealment.

• Allocation concealment was done after the participants were contacted by an independent centre which held the sequence list (Monami et al., 2015; Lincoln et al., 2008).
• Allocation concealment was done by the generation of the last digit of the participants' SSN (Malone et al., 1989).
• No further information was provided to state whether the envelope was opaque or sealed before allocating it to the groups (Gershater et al., 2011).

2 studies have an unclear allocation concealment (Rönnemaa et al., 1997; Bloomgarden et al., 1987) as no information was provided.

#### 4.2.3 Blinding (performance bias and detection bias)

Outcome assessment was masked in Gershater et al. (2011) and Lincoln et al., (2008).

#### 4.2.4 Incomplete outcome data

The withdrawal/dropout rate was high in Gershater et al. (2011), while Lincoln et al. (2008) study conducted an intention-to-treat (ITT) analysis for the primary outcomes.

#### 4.2.5 Selective reporting

There were no reports of the study protocol however the trial report lists the outcomes of interest in both the methods and result sections (Manomi et al., 2015; Gershater et al., 2011; Lincoln et al., 2008; Malone et al., 1989).

The information about the study protocol was unavailable. The outcomes (incidence of ulcer and amputation, callus, nail problems, infections due to fungi and behaviour assessment score) were not specified in the method section of the study (Bloomgarden et al., 1987).

#### 4.2.6 Other bias

• Co-interventions were not described and no data for adherence was provided (Gershater et al., 2011; Malone et al., 1989).
• Co-interventions were not described and data for adherence was provided (Bloomgarden et al., 1987).
• Co-interventions included regular podiatry care and suitable footwear when appropriate, but no structured education (Lincoln et al., 2008). Co-interventions such as podiatry care was provided to only the intervention group (Rönnemaa et al., 1997)

### 4.3 Risk of foot ulceration of study participants in the included studies

In four of the six included RCTs, participants were at high risk of diabetic foot ulceration (Monami et al., 2015; Gershater et al., 2011; Lincoln et al., 2008; Malone et al., 1989) while in two RCTs participants were at low or medium risk of diabetic foot ulceration (Rönnemaa et al., 1997; Bloomgarden et al., 1987).

### 4.4 Different approaches of patient education used in the included studies

Two RCTs focused solely on patient education in diabetic foot care (Monami et al., 2015; Gershater et al., 2011). In Gershater et al. (2011), the patient education was held for an hour. The intervention was compared to the standard information based on the international consensus on diabetic foot provided by diabetes specialist nurses. In Monami et al. (2015) the intervention group had a 2-hour (90 minutes interactive session on practical exercises on behaviour modification and 30 minutes face-to face lessons on risk factors in developing DFU) education program in a group session of 5–7 participants. The intervention used a physician for 15 minutes and a nurse for 105 minutes. Patient Interpretation of Neuropathy (PIN) questionnaire was administered before and after the educational session. This intervention was compared to brief leaflet information on foot ulceration, as suggested by local protocols.

In assessing the effectiveness of the patient education on diabetes education combined with an aspect of foot care as an intervention, Bloomgarden et al., 1987 compared it with usual care provided. In Bloomgarden et al. (1987) the content of the educational program comprised of 9 group sessions in which one of the session focused on foot care and skin hygiene.

While in the other five RCTs, an intensive foot care educational approach was compared with a less proactive foot care intervention (Lincoln et al., 2008; Rönnemaa et al., 1997; Malone et al., 1989). The interventions in these studies were different. In Lincoln et al. (2008) and Malone et al. (1989), the patient education was held for an hour and reinforced using written instruction. While in Malone et al. (1989) this intervention was compared with routine patient education in the control group while Lincoln et al. (2008) this intervention was compared with written instructions only. In Rönnemaa et al. (1997) the intervention group had 45 minutes individual patient education on proper foot wear and hygiene combined with variable number of follow-ups at a podiatry clinic and was compared to written instructions on foot care only.

## 5 Duration and follow-up

The follow-up period ranged from 6 months (Monami et al., 2015) to 7 years (Rönnemaa et al., 1997).

### 5.1 Themes within the review

#### 5.1.1 Effectiveness and efficacy of intervention (education) in preventing/reducing DFU and amputation rates

The primary outcomes of interest were preventing DFU/recurrence and amputation rates after educational intervention. The intervention in each study was education which was compared with standard education or different approach (written instruction, no intervention, and brochure). Data for diabetic foot ulceration with (42% (n = 5)) was provided by (Monami et al., 2015; Gershater et al., 2011; Lincoln et al., 2008; Malone et al., 1989; Rönnemaa et al., 1997). While the data for the amputation rate with (17 % (n = 2)) was provided by Malone et al. (1989) and Lincoln et al. (2008).

### 5.2 Results of meta-analysis/effect estimates and heterogeneity

#### 5.2.1 Education (diabetes and foot care education) versus usual care

**Fig. 2:**
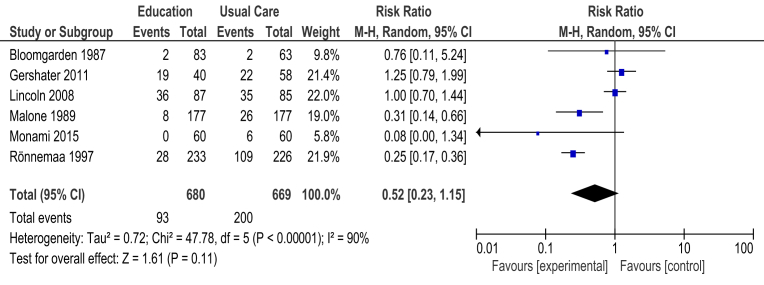
Forest plot of comparison: Comparison 1: Education (diabetes and foot care education) versus usual care.

Six RCTs had incidences of diabetic foot ulcers as an outcome. The data from (Monami et al., 2015; Gershater et al., 2011; Lincoln et al., 2008; Rönnemaa et al., 1997; Malone et al., 1989; Bloomgarden et al., 1987) were combined using a random model effect to compare education (diabetes and foot care education) versus usual care (Fig. 2). Meta-analyses were carried out for the outcome: incidence of diabetic foot ulcers and findings were based on 1,203 study participants.

#### 5.2.2 Intensive versus brief educational intervention

**Fig. 3:**
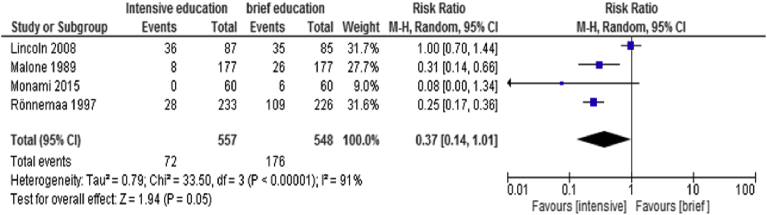
Forest plot of comparison: Comparison 2: intensive versus brief educational intervention.

Data from 4 RCTs (Monami et al., 2015; Lincoln et al., 2008; Rönnemaa et al., 1997; Malone et al., 1989) were combined using a random model effect to compare intensive versus brief educational intervention. Fig. 3 indicates an RR 0.37 (95%CI 0.14, 1.01) (P = 0.05) with high heterogeneity of 91% to assess for the incidence of DFU.

### 5.3 Amputation rates

**Fig. 4:**
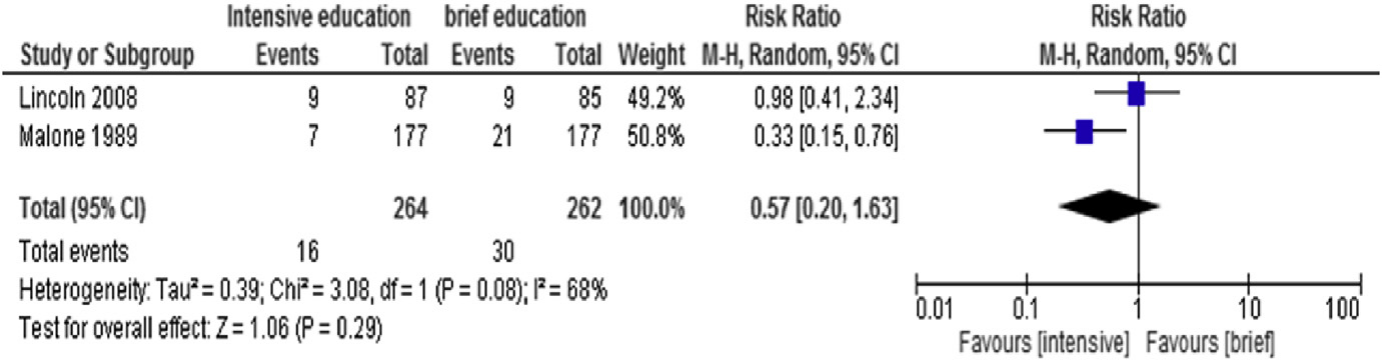
Forest plot of comparison: Comparison 3: intensive versus brief educational intervention.

Data from 2 RCTs (Lincoln et al., 2008; Malone et al., 1989) were combined using a random model effect to compare intensive versus brief educational intervention to assess for amputation rates in diabetic patient with high risk of foot ulceration. Fig. 4 demonstrates an RR 0.57 (95%CI 0.20, 1.63) (P = 0.29) with moderate heterogeneity of 68%.

## 6 Discussion

This review explored the effectiveness of education, its impact in the prevention of DFU and amputation rates in comparison with usual care or other control intervention strategies. Analysis based on intensive versus brief educational approach showed a statistically significant effect (P = 0.05) with a reduced risk of incidence of DFU in the intervention group when compared to the control group. The result had a high heterogeneity of 91% which demonstrated the variation in the studies. Rönnemaa et al.'s (1997) seven-year study explored how an intensive 45 minutes education program comprised of discussions on proper foot wear/hygiene, combined with podiatry care, was compared to written foot care instructions. Whilst, Malone et al.'s (1989) study compared an intensive hour group education on foot care by a podiatrist with routine foot care education. The median interval between instruction and follow-up measurement ranged between 1 to 26 months. Both studies were judged to have a high risk of incomplete data which may have affected their findings. Further bias was observed with regards to randomisation and allocation concealment in Rönnemaa et al. (1997). However, the randomisation method remained biased as it used an odd/even last digit of the participants' SSN, unlike Lincoln et al. (2008) study judged to have a low risk of bias in all the six domains in the assessment of the quality of the study. The study compared an hour intensive group education with written instructions.

This review also demonstrated that there was inadequate robust evidence to establish education alone can prevent/reduce DFU and amputation rates. Many studies reported the beneficial effects of education in improving foot care knowledge and self-care practices in those with DM. However, due to the lack of a standardised assessment tool, it was difficult to conclude the effects of education in improving foot care knowledge and self-care practices. The findings are in line with Dorresteijn et al.'s (2014) review that reported there was insufficient evidence to demonstrate limited education alone can prevent DFU and amputation rates.

This review's findings are in contrast with Némcova and Hlinkova's (2014) and Fujiwara et al.'s (2011) studies that reported overall effectiveness of education in preventing/reducing DFU. However, Fujiwara et al.'s (2011) uncontrolled before/after interventions could have provided decisive evidence of the effects of the diabetes foot care education program DFU. Yet the lack of a control group makes it hard to assess the reliablity of the findings (Fujiwara et al., 2011). The study could also have benefitted from a crossover trial or a staggered intervention group design to confirm the significance of the effectiveness of the program, especially in transferring the knowledge to other settings (Fujiwara et al., 2011). On the other hand, Némcova and Hlinkova's (2014) study on the efficacy of diabetic foot education focused on health promotion strategies on DFU prevention. Arguably, the study would have gained a broader understanding of diabetic foot care education by using a qualitative approach to explore issues which impact upon patient education (patients' values and views) and by applying an interpretative research strategy.

As previously stated, DFUs are a common occurrence in about 15% of diabetic patients with peripheral neuropathy, with complications such as deep infection, abscess, and osteomyelitis (Cychosz et al., 2016) and are a major worldwide health care concern. As a consequence of these complications, diabetes patients with recurrent plantar pressure foot ulcers, for example, have been estimated to require amputation in 71%–85% of cases (Laborde, 2008). In excess of 60% of non-traumatic lower limb amputations occur in diabetic patients, and pressure ulcers are the causative factor in up to 84% cases Centers for Disease Control and Prevention (2004). Ulcerations of the plantar aspect of the foot are frequently correlated with peripheral neuropathy, vascular disease, and elevated local pressure under the metatarsal heads due to a plantar flexion deformity of 1 or more of the metatarsal bones and associated lesions have been linked with higher rates of depression and lower quality of life for diabetes patients (Biz et al., 2018). Thus, the introduction of effective patient education programmes in preventing and reducing the incidence and/or recurrence of adult diabetes (plantar) foot ulcers and associated complications is imperative.

## 7 Conclusion

The importance of foot care education practices for diabetic patients, who are at risk of DFU and amputation, remains a core part of diabetes patient education. This review aimed to assess the effectiveness of educational intervention in preventing DFU and amputation rates. The outcome measures were to assess the beneficial effects of education in reducing DFU/amputation and improving foot care knowledge and self-care practices. Six RCT's met the inclusion criteria for this systematic review from health care databases and other sources. Overall, an intensive education approach offered a positive result in the reduction of DFU in the short term.

## Declarations

### Author contribution statement

Priscilla Adiewere, Gary Adams: Conceived and designed the experiments; Performed the experiments; Analysed and interpreted the data; Contributed reagents, materials, analysis tools or data; Wrote the paper.

Richard B. Gillis, Shahwar Jiwani, Andrew Meal, Ian Shaw: Analyzed and interpreted the data; Contributed reagents, materials, analysis tools or data.

### Funding statement

This work was supported by the Independent Diabetes Trust (IDDT).

### Competing interest statement

The authors declare no conflict of interest.

### Additional information

No additional information is available for this paper.
